# Editorial: Global excellence in emotion regulation and processing: Europe

**DOI:** 10.3389/fnbeh.2023.1138976

**Published:** 2023-01-31

**Authors:** Luísa Pinto, Francesca Calabrese, Patrícia Patrício

**Affiliations:** ^1^Life and Health Sciences Research Institute (ICVS), School of Medicine, University of Minho, Braga, Portugal; ^2^ICVS/3B's—PT Government Associate Laboratory, Braga, Guimarães, Portugal; ^3^Behavioral and Molecular Lab (Bn'ML), University of Minho, Braga, Portugal; ^4^Department of Pharmacological and Biomolecular Sciences, Università degli Studi di Milano, Milan, Italy

**Keywords:** emotion, mood disorders, brain processing, animal models, human studies

To understand emotion regulation and processing is to understand what makes us conscious humans, what drives our impulses and motivations. It also sets the stage to study how the underlying neural systems can be deregulated and become actionable targets for the development of therapeutic approaches to treat mood-related disorders.

This Research Topic gathers six high-quality works from research teams across Europe to advance the study of emotion regulation, from the fundamental and preclinical research to human experimental research and technological development.

Animal models are a cornerstone in the study of brain processing and emotion regulation in particular. Scientists have long developed, optimized and refined animal models to produce information on how the brain processes environmental stimuli, either positive or negative, and on how peripheral body changes and brain processing, bidirectionally interfere with each other, and not seldom, impact on emotion. For instance, in the last years researchers have shed light on the bidirectional communication between the brain and the gut, with a particularly relevant role for the immune system as a mediator (Ratsika et al., 2023). In this Research Topic, Escudero-Lara et al. implement a non-surgical mouse model that recapitulates the symptoms of human endometriosis, a chronic inflammatory disease that presents important emotional manifestations and that affects 10% of women in reproductive age. These mice developed abdomino-pelvic hypersensitivity, cognitive deficits, anxiety and depressive-like behaviors, which were accompanied by increased levels of inflammatory markers in brain areas including, periaqueductal gray, medial prefrontal cortex and hippocampus. The authors also investigated the role of CD4+ cells in the endometriosis-associated phenotype, using a CD4+ cell-depleting antibody, and observed a specific decrease in the anxiety-like behavior, that did not correlate with diminished mechanical hypersensitivity. This study reinforces the role of inflammatory response on emotional regulation.

A second study, Brivio et al. used a 5-HTT knock out rat model in a fear conditioning and extinction paradigm to investigate the role of serotonin in mitochondrial function in the context of stress-related disorders. In a previous study, the dynamics of these organelles have been shown to be altered after stress (Brivio et al., 2022), one of the most important precipitating factors for depression and other psychiatric disorders. Here, Brivio et al. show, for the first time, that the fear extinction recall impairment observed in 5-HTT–/– rats is accompanied by alterations of mitochondrial dynamics in the amygdala and prefrontal cortex. This study, identifies these two brain regions, involved in memory, learning and threat sensitivity, in the link between stress vulnerability, mitochondria and the serotonergic system.

Anorexia nervosa (AN), a psychiatric disorder that encompasses an important emotional instability component, is the focus of the study by Mottarlini et al., also featured in this Research Topic. The authors studied the amygdala, due to its role in emotion processing and show that altered amygdala BDNF might be critical for AN vulnerability traits.

Analyzing complex social interactions may be crucial when studying emotion regulation in many neuropsychiatric disorders and their neurobiological correlates. Though, in preclinical settings this is often overlooked, as most laboratory rodents are housed in small numbers and in modestly enriched environments, and social interplay is typically assessed in one-way interactions in short behavioral paradigms. This is the main topic addressed in the study of Amorim et al. The authors longitudinally analyzed how social interactions are affected by food accessibility and by conditioned access to a female rat, in a colony of six rats living in a previously validated enriched environment (Castelhano-Carlos et al., 2014). By selectively controlling a number of variables, the authors showed how the availability of resources modulates social behavior both at group and individual levels, revealing the construct value of the model for studying the neurobiological foundations of this behavior.

Research has demonstrated relevant associations between pain, long-term stress exposure, and neuropsychiatric disorders (Schatzberg, 2004). In the field of emotion regulation and processing, where specificities and idiosyncrasies of human behavior must be considered, experimental research with human subjects is essential to advance and complement translational and preclinical research. Pain chronicity which has been linked to trauma and trauma-related disorders, has been suggested, both by empirical research and clinical observations, to alleviate feelings of guilt, an important part of the psychological reaction to trauma. In an attempt to test this hypothesis, while validating a method of guilt-induction through a written auto-biographical essay, Schär et al. developed an experimental study with healthy male participants, as presented in this Research Topic. Their results show no impact of pain on behavioral guilt-ratings and question previous works proposing an impact of pain on moral emotions.

Emotion regulation research in humans is often accompanied by the collection of physiological signals, such as heart rate and general activity, through biosensors. These allow the assembly of large amounts of real-world data that may be used to advance research (Johnson and Picard, 2020). In this Research Topic, de Looff et al. describe the generation of an R package to process the signal data, offering flexible signal pre-processing, artifact detection, and feature extraction, with a built-in visualization tool.

Together, in our opinion, these works represent a relevant collection of research covering different aspects of emotion regulation and processing using a panoply of diverse but complementary approaches.

## Author contributions

PP wrote the manuscript. LP and FC revised and approved the final version of the manuscript. All authors contributed to the article and approved the submitted version.
